# Mapping collective variable and synergy dynamics to task outcome in a perceptual-motor skill

**DOI:** 10.1371/journal.pone.0215460

**Published:** 2019-04-18

**Authors:** Yeou-Teh Liu, Kuo-Liang Chuang, Karl M. Newell

**Affiliations:** 1 Department of Athletic Performance, National Taiwan Normal University, Taipei, Taiwan; 2 Department of Physical Education, National Taiwan Normal University, Taipei, Taiwan; 3 Program of Athletic Performance, National Taitung University, Taitung, Taiwan; 4 Department of Kinesiology, University of Georgia, Athens, Georgia, United States of America; Purdue University, UNITED STATES

## Abstract

In complex adaptive systems approaches to perceptual-motor control the mapping between the different categories of task dynamics: namely, task outcome, collective variable, neuromuscular synergies and individual joint configurations is a central theoretical issue, that has been primarily studied in bimanual tasks. Here we report an investigation in the roller ball task of how the task goal and multiple degrees of freedom of the arm-hand complex affords degeneracy between the respective properties of the task dynamics. The relation of the candidate collective variable, namely, the synchrony of the inner ball to outer shell motion of the roller ball and its relation to the task goal (continued increasing in ball speed), was examined as a function of the initial ball speed acting as a control parameter. Within trial analysis revealed initial search behavior for synchrony of ball and shell motion that was longer in duration with initial lower ball speed conditions. In contrast, higher initial ball speed conditions reduced the search time for and enhanced the rate of stabilization of the synchrony of inner ball and outer shell motion–features that facilitated the continued increase of ball speed and the probability of task success. Participants adopted one of three wrist-elbow neuromuscular synergies to manipulate the roller ball, the distribution of which was not influenced by either initial ball speed or task outcome. The pattern of findings over the different properties of task analysis of the roller ball provides evidence for the distinct but complementary dynamics of searching to form, stabilize and exploit a collective variable that satisfies the task goal through a small redundant set of arm-hand synergy motions.

## Introduction

A contemporary umbrella perspective to perceptual-motor control is that of complex adaptive dynamical systems [1–2]. There have been several theoretical and experimental instantiations in the perceptual-motor domain that reflect this perspective that have a number of common elements concerning movement coordination, control and skill (e.g., [3–6]). These include the system properties of multiple degrees of freedom (DFs), interacting subsystems within and between levels of analysis, emergence of movement coordination modes, and the exhibition of varying levels of the complexity of system output that continually evolve with learning and development over the lifespan [5].

Within this adaptive dynamical systems framework, a central theoretical and experimental strategy from coordination dynamics is the determination of the collective variable of the perceptual-motor system and the control parameter(s) that change the state (stability/instability) of the system. In the experimental paradigm of bimanual coordination increasing the frequency of oscillations of the two fingers induces a phase transition from an anti-phase to an in-phase coordination mode [7–8]. This foundational phenomenon supports the dynamical interpretation of frequency acting as a control parameter and relative phase of the two fingers reflecting an order parameter or collective variable in the Haken-Kelso-Bunz (HKB) model [7].

There have been many experimental investigations of the dynamics of the bimanual paradigm [9–10] with a few theoretical elaborations [11–12] but the essential properties of the HKB model have been preserved. However, there has been almost no investigation of the collective variable dynamics outside of the various bimanual paradigms. One limitation of the original bimanual finger wiggling paradigm is that the collective variable (relative phase of the motion of the two fingers) in essence also reflects the neuromuscular synergy for the task. There has emerged a significant theoretical place for synergies in motor control but with it there has been a broadening range of the definitions of a synergy (see [13–15]). Here, following Bernstein [41], we draw on the more traditional anatomical-neuromuscular interpretation of movement synergies as a collective of muscles given that the movement coordination of the arm-hand complex in the roller ball corresponds with these foundational movement units. In many perceptual-motor tasks, however, it can be anticipated that the collective variable will be on a dimension distinct from the neuromuscular synergy due to the flexibility in task dynamics arising from the multiple joint space DFs that are available for coordination and control. Moreover, in the bimanual paradigm of learning a new relative phase (e.g., 90 degrees [16]), the to-be-achieved relative phase is also the task goal leaving no degeneracy between the task outcome and the relative phase–a direct mapping that in our view is not present in most perceptual-motor skills.

In a series of recent experiments we have investigated the dynamics of learning and performing the roller ball task [17–19]. The roller ball is made up of an inner top that can spin about a rod within another outer ball-shaped casing that is held in the hand. The performer initiates the spinning of the inner top when holding the roller ball in one hand and tries to keep it spinning with the arm-hand motion. Inside of the outer casing, there is a grand circular groove that allows the two ends of the rod of the inner top to move along. When the inner top starts to spin about the rod, the angular momentum generated from the spin motion will push the rod moving along the circular groove. If a synchronous motion from the outer ball-shaped casing can be applied to the precessional motion of the inner top, it will enhance the speed of the inner top from the initial spin [20].

The roller ball, although generally considered a child’s game or an exercise task where keeping the inner ball moving at the same initial speed or greater for a period of time is considered a successful performance, has been analyzed for its mechanical principles [21–22]. Furthermore, we have shown that the roller ball is attractive as a perceptual-motor task for the study of motor learning and control because it requires a rich set of dynamics that depend on the tight relation between the haptic feedback and the movement of the rolling effector arm-hand complex to solve the task demand but it is not too complicated to decompose the dynamics thereof. The experiment presented here was set-up to map the distinctive properties of task dynamics and their relations, including, between the task outcome (ball speed and probability of success), the ball-shell synchrony (candidate collective variable) and the arm-hand motion synergy as a function of initial conditions (ball speed). A goal was to experimentally separate these task properties and in particular distinguish the candidate collective variable from the neuromuscular arm-hand synergies and task goal.

The arm-hand complex with its many DFs (muscles and joint configurations) affords a rich array of prehensile movements including established patterns of neuromuscular organization that support limb motions and their use in the actions of reaching and grasping [23–25]. There are neuromuscular synergies for the core motions of hand pronation/supination, wrist rotation and digit opposition that can be organized into functional adaptive movements in prehensile tasks. In our view, however, they do not represent the collective variable of the roller ball task or perceptual-motor skills more generally. One of our goals within the multiple variable approach to task dynamics was to distinguish the adaptation of the collective variable from the neuromuscular synergies used in support of executing the task.

Our previous studies have provided evidence for the initial conditions of ball speed and practice acting as dual control parameters in the roller ball task performance, with the S-shape increase in the probability of task success and learning modeled as a 1^st^ order non-equilibrium transition [17–19]. In general, participants have a greater probability of success in enhancing ball speed and performing the task the higher the initial ball speed. Higher initial ball speeds generate stronger precession motion of the ball [20–21], and the resulting movement of the rod along the grand groove on the outer shell, we hypothesize, provides the source of the haptic feedback that mediates arm-hand motion in performing the roller ball task. Practice leads to an enhanced probability of success in performing the task that is dependent on the initial ball speed conditions.

The roller ball is a task that tends to have an all or none functional character at the behavioral performance level in that individuals can in essence either perform the task (enhance ball speed) or not. The learning process of this type of task has been modeled using saddle-node dynamics where increasing practice time, which acts as the control parameter of the learning dynamics, may lead to a transition from failure to success in performing the task. Being on or around the critical point of the saddle-node dynamics produces an unstable performance [18–19]. Bicycle and unicycle riding [26] and hoola-hooping [27] along with the roller ball appear to fall into this type of task and that produce different but related functions of learning [19, 28] than the traditional accounts of learning movement scaling tasks as exponential and power law change [29], but they have been considerably less studied.

Our previous studies on the learning and performance of the roller ball tasks were determined on the basis of the probability of enhancing the ball velocity as a function of initial ball speed conditions and practice time. In other words, the dynamics of learning and performance were assessed only at the task outcome level (probability of success). Although the roller ball task is performed with the multiple joint space degrees of freedom of a single arm-hand kinematic chain, the task success we have conjectured requires the synchrony of the inner ball and outer shell motion–in essence making the collective variable functionally a 2-degree of freedom coupling task. The nature of the roller ball task also leads this hypothesized collective variable to be defined over the relation of the motion of the end effector (hand gripping the outer shell of the roller ball) and a property of the environment (the motion of the inner spinning ball). The collective variable can be distinct from the neuromuscular synergy in the task dynamics of perceptual-motor skills, such as bicycle riding and hula hooping, in part because it is defined over the individual, environment and task constraints [30–31].

We have proposed that the key dynamic property determining task success in the roller ball task is the synchrony of the inner ball motion to that of the outer shell motion [17–19]. And, furthermore, that the roller ball task affords a separation of the neuromuscular effector synergetic motions from the candidate collective variable (ball-outer shell synchrony). Thus, the roller ball task provides the potential of degeneracy [32] to the mapping of the dynamics of the roller ball degrees of freedom and the ball speed solution of the task outcome.

In summary, this experiment investigated in an adaptation paradigm the effect of the control parameter (initial ball speed) on the performance outcome of the roller ball task. The specific focus was to map the relations between the task outcome (ball speed and task success), the ball-shell synchrony (candidate collective variable) and the arm-hand motion synergies. We tested hypotheses derived from findings of our earlier studies [17–18] that: 1) task performance would be dependent on producing a synchronous motion of the outer shell to the inner ball supporting the proposition that this is the collective variable for the task; 2) higher initial ball speed would increase the likelihood of producing the task relevant inner ball to outer shell synchronous motion; 3) higher initial ball speed conditions would reduce the duration of the search behavior for the ball-shell synchrony within a trial before adapting into a stable task performance, and 4) that there is movement degeneracy between the synchronous ball and shell motions, the arm-hand synergies and the initial ball speed in producing a successful roller ball task. Thus, it was anticipated that the experimental roller ball task provides the context to map the adaptive conditions of a control parameter (initial ball speed) to the distinctive but related properties of task dynamics–task outcome, collective variable, neuromuscular synergies and joint space DF [33–34].

## Methods

### Participants

Sixteen healthy, adult volunteers (3 females) between 22 and 36 years of age participated in the experiment. All the participants had some but different levels of experience with the roller ball task so that they could successfully accelerate the roller ball at least for the 40 rps initial speed. The experimental procedures and the participation consent form were approved by the Research Ethics Committee of National Taiwan University. Each participant read and signed the consent form before taking part in the experiment, and was paid a small honorarium after completing the experiment.

### Apparatus

We customized a rollerball system with a commercially available roller ball (E-Neng Tech, Taiwan), a desktop computer running on the Windows OS, and a data collection program developed with the LabVIEW system design software (National Instrument, version 11). The rollerball consists of an inner ball (diameter of 6 cm) that is covered by a spherical outer shell (diameter of 7 cm) and can be held in one hand (see [17, 35] for detailed description of the roller ball device). The outer shell movement is directly manipulated by the arm-hand motion and therefore the movement characteristics of the outer shell is used to represent the effect of the arm-hand coordination. There is a 3.5 cm diameter circular opening on the outer shell of the roller ball that provides a direct contact to the inner top. Two fiber optic wires were inserted to the outer shell of the roller ball on the opposite side of the circular opening to detect the black and white differential light reflections from the inner top when it spins. The output of the fiber optics was sent to the desktop computer via a digital fiber amplifier (Riko, Taiwan, BR2-N) and a 16 bit A/D board (National Instrument, 6034E) at a sampling rate of 200 Hz. We tested the roller ball’s performance before the experiment. Only those roller balls that demonstrated a stable deceleration rate of 3 ± 0.4 rps/s from 40 rps initial speed to stop were used in the experiment [19].

A 4-camera, 200 frames per second (fps) video system (Jai Pulnix TM-6740GE, AZ: Aegis electronics; Stream pix 4.0, Montreal Canada: NorPix) was used to capture the 3-D movement of the outer shell (hand) and the precession motion of the inner ball that was visible through the opening of the outer shell. The cameras were synchronized to the roller ball system through a light signal on the computer screen that indicated the beginning of a trial.

### Task

The task was to accelerate the roller ball above the respective initial speed by the end of the 10 s trial. To start the ball spinning, a string was inserted into a hole in the inner ball and wrapped around the inner ball on the groove. The inner ball would start to spin when the string was pulled out of the hole. The harder the string was pulled, the higher the spinning speed. The spinning speed started to decrease after the string was out of the ball. The spinning speed was monitored on the computer screen and a warning signal followed by a go signal would alert the participant when the spinning speed reached the pre-set value for the specific initial speed condition. There were 5 initial-speed conditions: 40 rps, 30 rps, 20 rps, 15 rps, and 10 rps, and 10 blocked trials were performed at each initial-speed condition. All participants performed the task in the same order of 40 rps, 30 rps, 20 rps, 15 rps, to 10 rps initial-speed. Although participants were in general encouraged to accelerate the roller ball as fast as possible as soon as they heard the go signal, they were informed not to continue to accelerate the roller ball for the trial if the spinning speed exceeded 60 rps due to the limitation of the system.

### Procedures

Because there are many different ways to hold the roller ball and many different movement patterns to accelerate the roller ball, the participant was required to demonstrate the way he/she performs the roller ball before we set up the positions/orientations of the cameras. One reflective marker was attached to the edge of the circular opening of the outer shell. The participants were asked to hold the roller ball in such a way that would not cover the marker and the circular opening when they perform the roller ball task at all times. All the participants were able to perform the task under these requirements without any difficulty.

**Fig 1:**
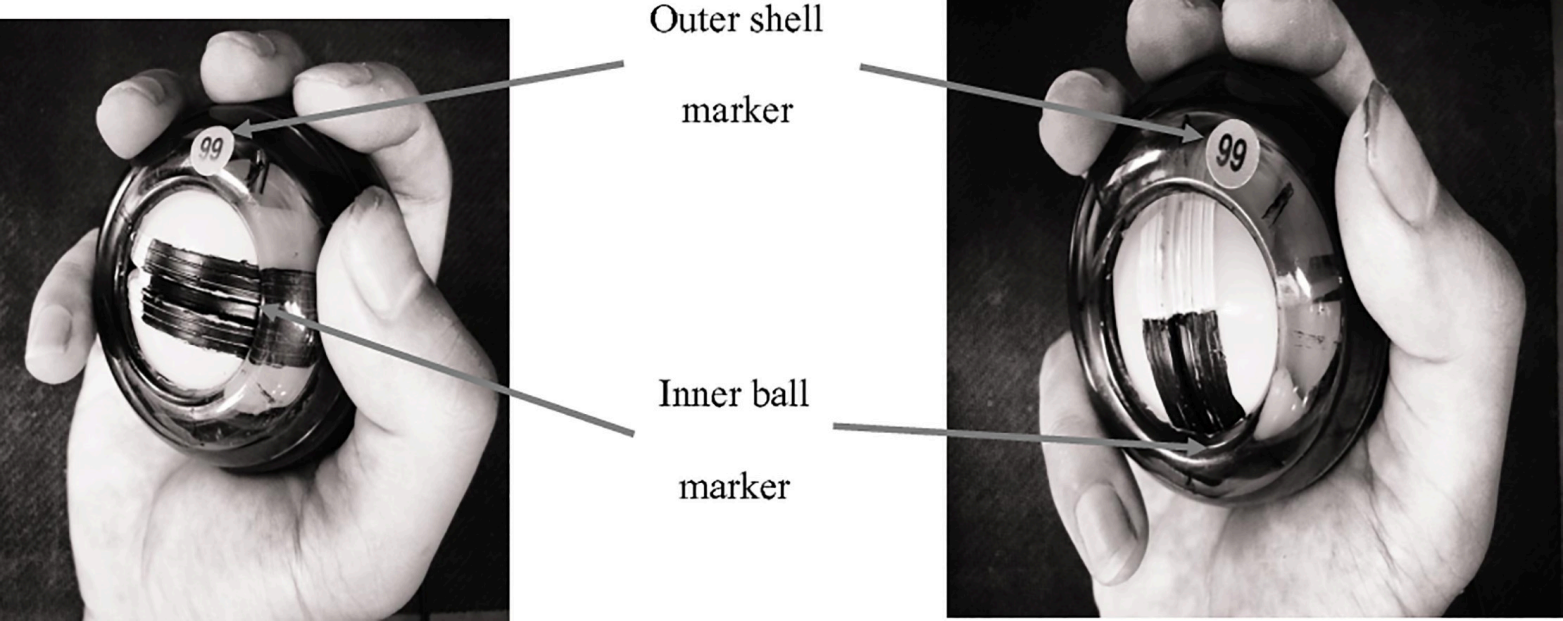
Illustrations of the marker locations of the outer shell and the inner ball of the roller ball.

After the participant demonstrated her/his individual roller ball movement comfortably and consistently, the cameras system was set up in such a way that the circular opening of the outer shell was in the viewing area of at least 2 cameras at the same time. This arrangement facilitates the reconstruction of the 3-D kinematics of the marker on the outer shell as well as the rotation position of the inner ball (see Fig 1). A painted groove rotated around the circular opening and the rotational position of the inner ball was identified by the intersection of the painted groove and the edge of the circular opening. The experimenter started spinning of the roller ball above the designated initial speed by pulling the inserted string out of the hole in the inner ball and then handed the roller ball to the participant.

The computer screen showed the current spinning speed of the roller ball and an alert signal would light up at 10 rps above the designated initial speed (for example, 50 rps for the 40 rps condition) to remind the participant to get ready to begin the trial. Another alert signal would sound when the roller ball speed descended to the designated initial speed and the participant would start to accelerate the roller ball. Ten seconds after the start audio signal, two different audio signals indicated whether the roller ball speed was above the initial speed or not. This end-point signal provided an outcome feedback of the trial for the performer.

### Data analyses

**Fig 2:**
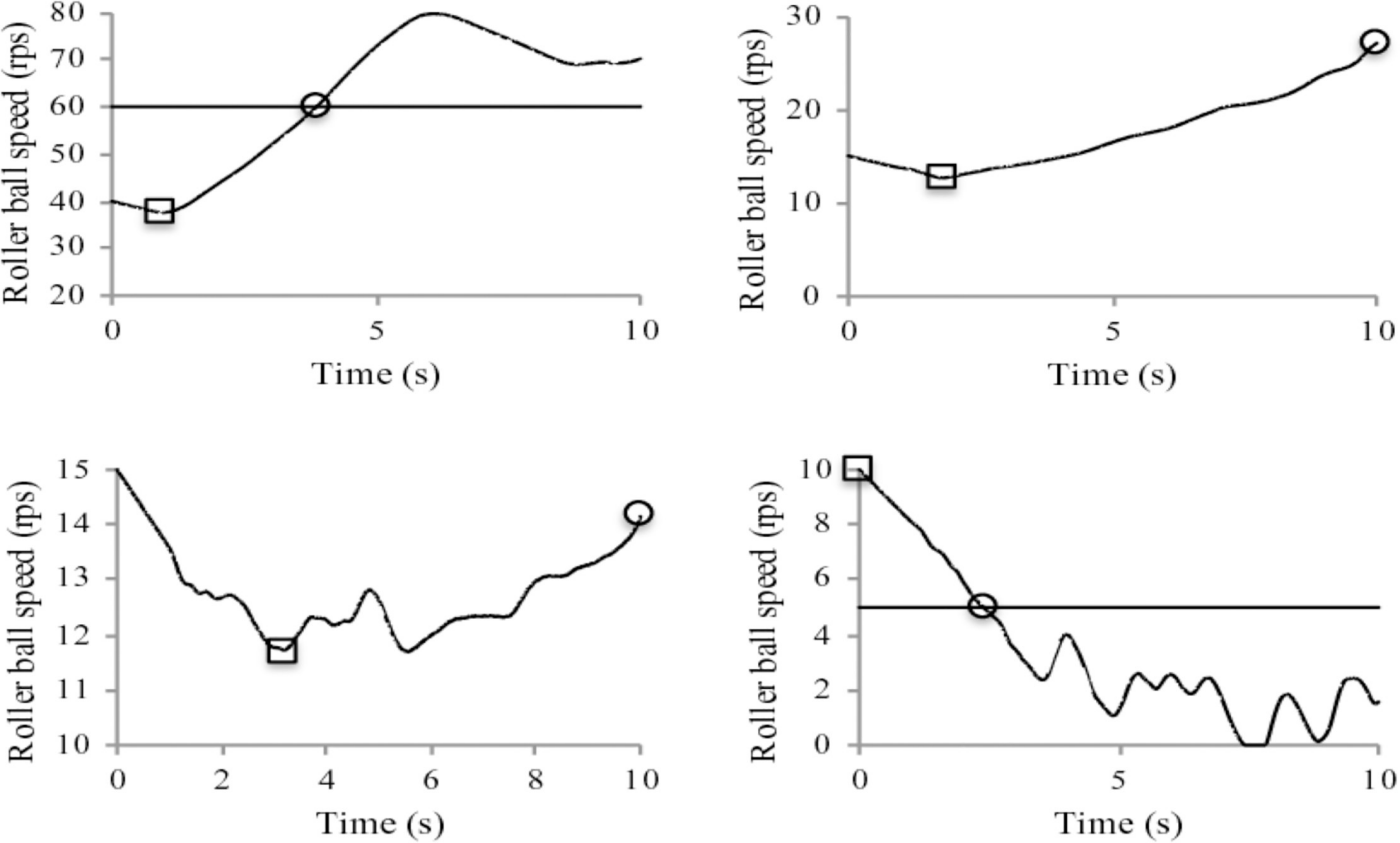
Example roller ball speed profiles and the identification of the beginning and end of the slope calculation of individual trials. The 10 s trial starts when the rollerball speed reached the initial speed of the condition. The square markers represent the beginning and the circle markers indicate the end of the slope calculations.

The roller ball performance outcome was analyzed in terms of the number of success and failure trials and the slope of the roller ball speed profile within a trial for each initial speed condition. A trial was considered as a success when the roller ball speed was above the initial speed at the end of the 10s trial. A simple linear slope was calculated from dividing the speed difference by the time duration between the start and end points (Fig 2). The Chi-squared test of independence was used to examine the association between the outcome of the roller ball trials and the initial speed condition. A one-way (5 initial speed) ANOVA with repeated measure was used to examine the slope of the speed profile.

In addition to assigning the result of success and failure to each trial, the slope of the roller ball speed profile in a trial provides a fine-grained measure of the trial performance. In general, a positive slope would result from the continuous increase (up to 60 rps due to the limitation of the equipment) of the ball speed after the latent period, and if the ball speed continued to decrease would result in a negative slope. When the ball speed decreased below 5 rps, there were occasionally random fluctuations that did not result in a persistent increase of ball speed [19], therefore, the time when the ball reached 5 rps was used as one criterion to calculate the end point of the slope. Using the lower and upper bound of the speed to calculate the slope also eliminated the floor and ceiling effect of the measurement system and provided a more accurate evaluation on the roller ball performance under the task constraints. The search duration, which is the time period of the initial decrease in ball speed that includes the time to the “go” signal and the exploratory motions to increase the ball speed, was defined as the time period from the start of the trial to the initial increase of the ball speed. Ten seconds would be registered as the search duration if the ball speed continued to decrease until the end of the trial. The one-way (5 initial speed) ANOVA with repeated measure was used to analyze the search duration. Fig 2 illustrates the start and end points on the example ball speed profiles for calculating the slopes. The average slopes over the initial speed conditions were fitted with the 4-parameter sigmoid function [18].

The arm-hand movements of the roller ball task were observed and categorized as the first step to describe the movement patterns of the roller ball task. Two observers who were experienced with the roller ball tasks established the general categories of the movement patterns based on the extensive viewing of the videotapes of the experimental tasks. A general principle for categorizing the movement patterns was established and used as the basis for categorization. Twenty percent of the total 800 trials were randomly selected for intra- and inter-observer reliability tests. One week after the first categorization of the arm-hand motions the observers performed the categorization of the roller ball movement patterns again. Cohen’s Kappa was used to evaluate the intra- and inter- observer reliability, and the Kappa values ranged from 0.85 to 0.95.

Due to the labor-intensive digitization work, the kinematics of the outer shell and the inner ball of the roller ball were digitized from the videos for a selected portion of the trials. The selection principle was as follows: every participant had at least one trial randomly selected for each initial speed condition. If a participant had both success and failure trials in one initial speed condition, at least one successful trial and one failed trial were randomly selected for analysis. Qualitatively different performance within the same outcome group of a participant, for example, barely made the criterion of success vs. easily passing the criterion with continuous acceleration, also added another trial for analysis. Overall, there were 113 trials selected from 800 total trials, which included 16 trials from the initial condition of 40 rps, 20 trials from 30 rps, 28 trials from 20 rps, 32 trials from 15 rps, and 17 trials from 10 rps, used for analyzing the inner ball-outer shell kinematics of the roller ball movement.

The outer shell-inner ball movement videos were digitized using video digitizing software Simi Motion 9.0.6 (Simi Reality Motion Systems GmbH, Germany). The digitized data were smoothed with the 20 frames (0.1 s) moving windows. The resultant velocity calculated from the markers on the outer shell and the inner ball were used in the Fast Fourier Transformation (FFT) to obtain the dominant frequencies of the inner ball and outer shell motions. A matching frequency trial was identified when the dominant frequencies of the inner ball and outer shell motions were the same. A one-way (5 initial speed) ANOVA was used to analyze the outer shell dominant frequency, which was determined by the greatest power within the spectrum. Another one-way (3 movement synergy) ANOVA was also used on the outer shell dominant frequency. The resultant speeds of the inner ball and outer shell motions were also used in the Hilbert transform [36–37] to calculate the relative phase over the 10 s trials. Relative phase measure falls under the category of angular (circular) data, the summary statistics such as mean and standard deviation are not appropriate for angular data. Information entropy measures the probability distribution of the data. Information entropy of the relative phase was used to identify the successful trials where synchronous movement between the outer shell and the inner ball resulted in a fixed (concentrated) relative phase therefore a lower information entropy. Information entropy [38] was calculated for the relative phase of each trial with bin size of 10 degrees over 360 degrees.

The 113 selected trials were divided into the success and failure outcomes. The Chi-squared test of independence was used to examine the association between the outcome of the roller ball trials and the matching frequency trials. In addition, information entropy of the relative phase was examined using paired *t* test to compare the relation between the outer shell and inner top for the success vs. failure trials. For the repeated measure ANOVA, the Greenhouse–Geisser method was used to correct for violations of sphericity. The statistical significance level was set at α = .05.

## Results

### Roller ball outcome

The Chi-squared test for independence showed that the outcome of the roller ball task had a significant association with initial speed conditions, X^2^_4_ = 49.98, *p* < .01, Cramer’s *V* = .79. Table 1 shows the cross tabulation of the outcome and the initial speed conditions. In general, the number of success trials increased as a function of initial ball speed condition.

**Table 1:** Cross tabulation of the outcome and the initial speed conditions.

		Initial speed condition (rps)					
Outcome		10	15	20	30	40	Total
Failure	Count	16	9	4	0	0	29
	Percentage	20	11.25	5	0	0	36.3
	Adjusted residual	5.9	1.9	-1.0	-3.4	-3.4	
Success	Count	0	7	12	16	16	51
	Percentage	0	8.75	15	20.0	20.0	63.7
	Adjusted residual	-5.9	-1.9	1.0	3.4	3.4	
Total	Count	16	16	16	16	16	80
	Percentage						100

The slope of the roller ball speed profile in a trial provides a fine-grained measure of the trial performance. There was a time gap between the “go” signal and the start of the movement and so there was a brief dip of the roller ball speed before the rolling movement had an effect on the roller ball speed. Furthermore, there was the remainder trial time of 10 s for the participant to increase the roller ball speed to reach the required speed criterion. It is possible that although the roller ball speed increased following the first dip the increasing rate was not steep enough to reach the criterion speed at the end of the 10 s trial. In this case, the slope may be positive, but the trial is considered a failure. It is also possible that the roller ball speed did not increase, but the participant managed to slow down the ball deceleration leading to a failure trial and a flatter negative slope.

Examples of speed profile of individual trials are depicted in Fig 2. The one way repeated measure ANOVA result showed a significant ball speed effect on the slope, *F*(4, 60) = 100.23, *p* < .01, η_p_^2^ = .87. The post hoc comparisons revealed significant differences for all pairs of conditions except for the 30 and 40 rps comparison.

For the search duration analysis, the ANOVA showed a significant initial speed effect, *F*(1.38, 20.71) = 64.33, *p* < .01, η_p_^2^ = .81. Paired comparisons revealed that the durations for the 40 rps, 30 rps, and 20 rps were not different from one another, but they were all significantly shorter than those of the 15 rps and 10 rps conditions. In addition, the search duration for the 15 rps condition was significantly shorter than that of the 10 rps condition. Because the search duration was defined as the time from the start of the trial to the time when the arm-hand movement effectively increased the ball speed, the search duration for most of the failure trials was registered for 10 s.

**Fig 3:**
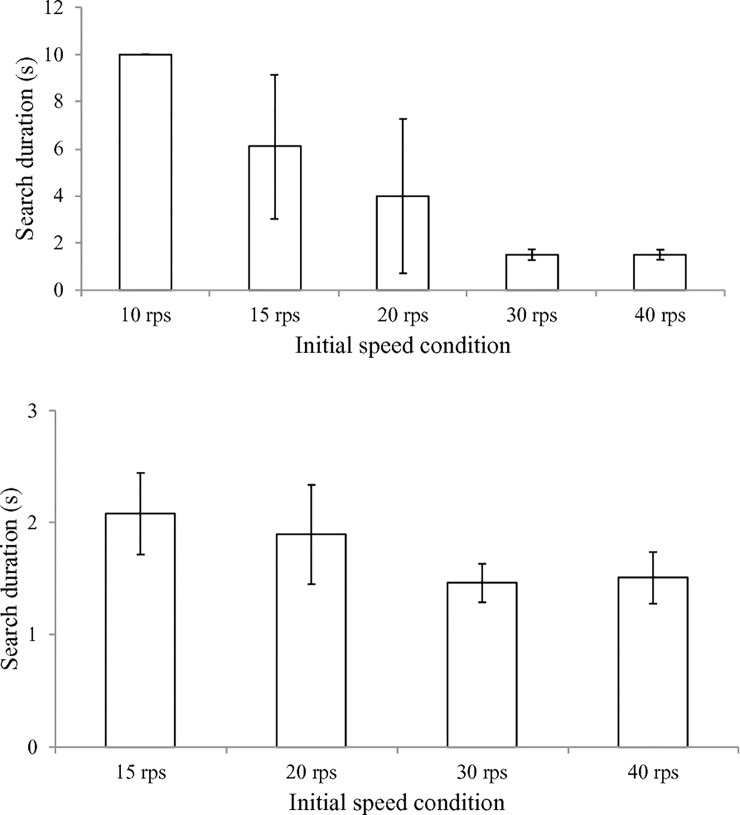
Average search duration for the initial speed condition over 16 participants (top panel) and average search duration of the success trials for the initial speed condition (bottom panel).

**Fig 4:**
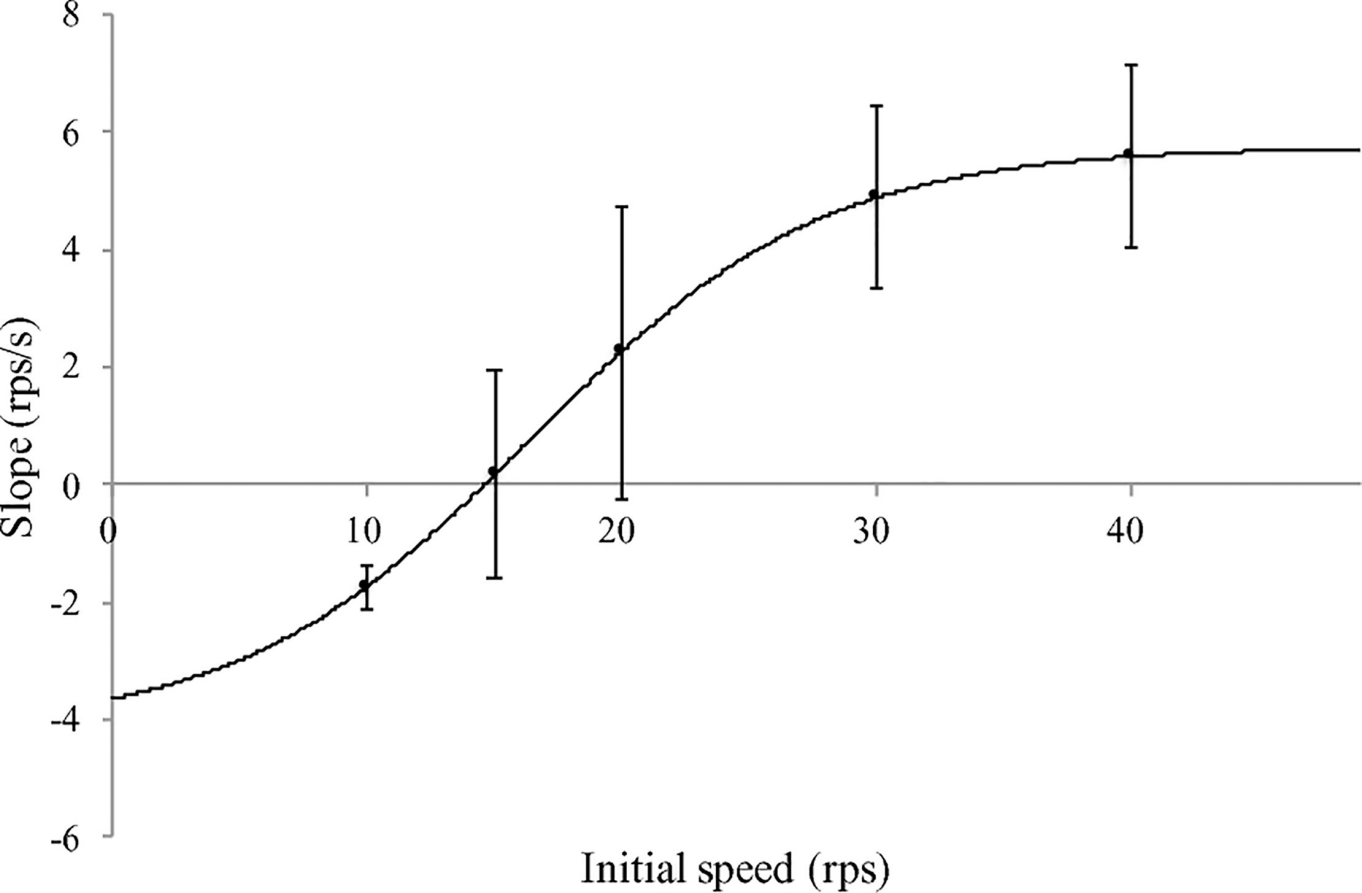
The sigmoid function fit of the average slope over 5 initial speed conditions. The error bars indicate the between participants SD.

An additional analysis on the success trials only was implemented for the 4 initial speed conditions (there were no success trials in the 10 rps condition). The ANOVA result showed a significant initial speed condition effect on the search duration of the success trials, *F*(3, 33) = 20.20, *p* < .01, η_p_^2^ = .65. Paired comparisons revealed that the search durations for the successful 40 rps and 30 rps trials were significantly shorter than those of successful trials in the 15 rps condition, and the search duration of the successful 30 rps trials was also significantly shorter than that of the 20 rps condition (*ps* < .01) (Fig 3). The average slope over the 16 participants for each initial speed condition was also fitted with a sigmoid function: ${f=y}_{0}+\frac{a}{1+e^{-\frac{x-x_{0}}{b}}}$ where y_0_ represents the lower bound of the slope, a is the difference between the lowest and the highest slopes, x_0_ is the inflection point and b^-1^ is related to the rate of change. Fig 4 shows the mean and SD of the slopes for each initial speed condition over 16 participants and the sigmoid function.

### Candidate collective variable

The 113 trials selected for the roller ball movement analysis were divided into the success and failure results. The X^2^ cross-tabulation analysis showed the significant associations between the outcome of the trials and the matching of the dominant frequencies between the outer shell and the inner ball, X^2^_1_ = 35.13, *p* < .05, Cramer’s *V* = .56. Table 2 shows the cross tabulation of the outcome of the trials and matching of the dominant frequencies. The one way ANOVA on the outer shell movement frequency showed a significant initial ball speed effect, *F*(4, 108) = 5.13, *p* < .01, η_p_^2^ = .16. Paired comparison results revealed that the outer shell movement frequency in the 10 rps and 15 rps conditions was significantly higher than those in the 30 and 40 rps conditions, *ps* < .05.

**Table 2:** Cross tabulation of the outcome and matching of the dominant frequencies of the outer shell and inner ball motions.

		Frequencies		
Outcome		Different	Matching	Total
Failure	Count	29	11	40
	Percentage	25.7	9.7	35.4
	Adjusted residual	5.9	-5.9	
Success	Count	12	61	73
	Percentage	10.6	54.0	64.6
	Adjusted residual	-5.9	5.9	
Total	Count	41	72	113
	Percentage	36.3	63.7	100

**Fig 5:**
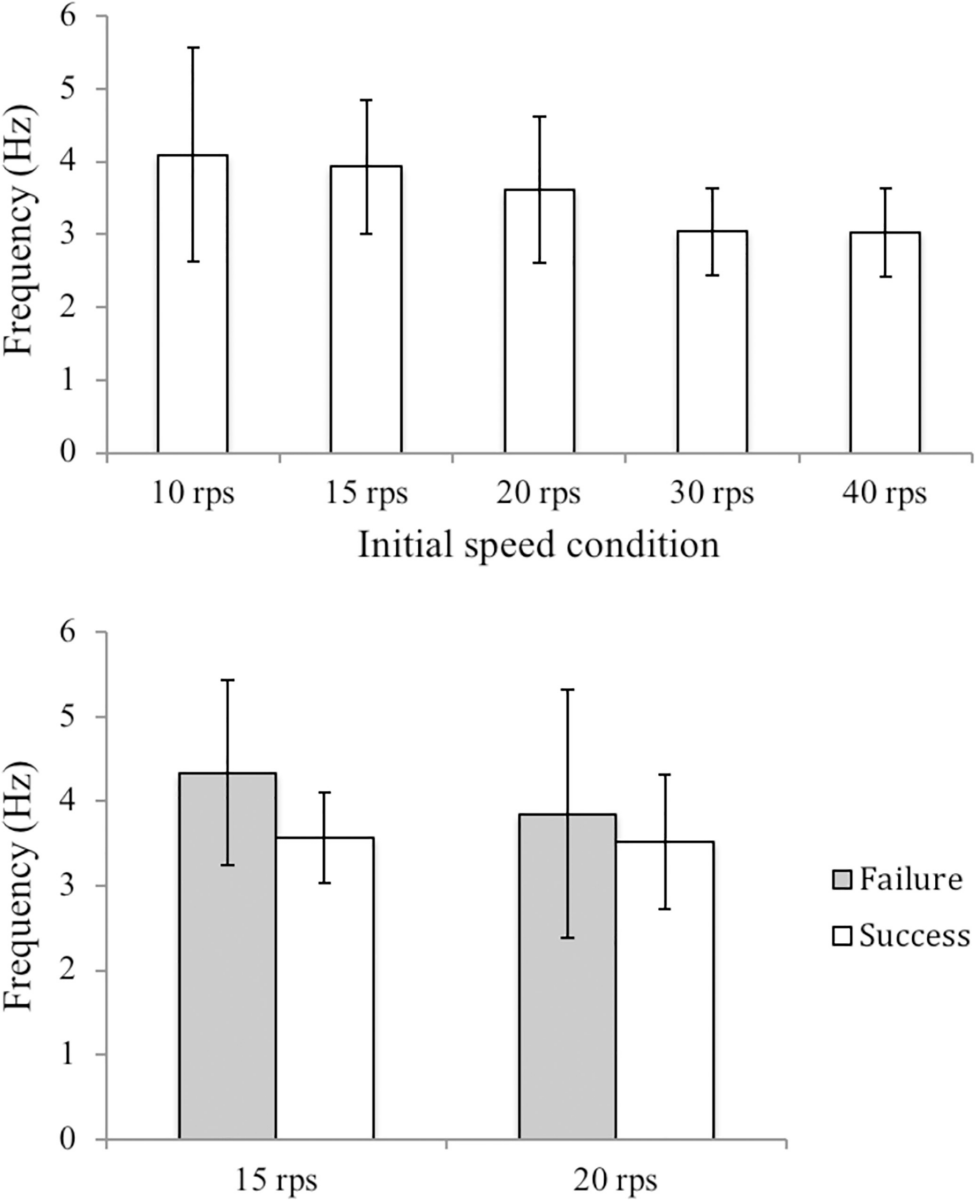
The top panel shows the average outer shell movement frequency of each initial speed condition from the 113 trials. The bottom panel shows the average outer shell movement frequency of the success and failure trials for the 15 rps and 20 rps initial speed conditions.

Further examining the difference of outer shell movement frequency between the success and failure trials, we used the independent sample *t* test on the 15 rps and 20 rps initial speed condition where both failure and success trials were found. The results showed a significant difference for the 15 rps initial speed condition where failure trials had a higher outer shell movement frequency than those of the success trials, *t*_*30*_ = 2.59, *p* < .05. No significant difference was found, however, for the 20 rps initial speed condition, *t*_*26*_ = .77, *p* = .45 (Fig 5).

Although the high association between the matching frequency of the outer shell and the inner ball was found in the success trials, we did not observe any particular positional value of the relative motion that was associated with the success or failure trials. This was expected because of the circular nature of the movement of the ball and the shell. Further examination of the relative phase of the ball and outer shell of the successful trials revealed that they tended to show a stable synchronous motion although the specific position of the ball to outer shell may be different between trials.

**Fig 6:**
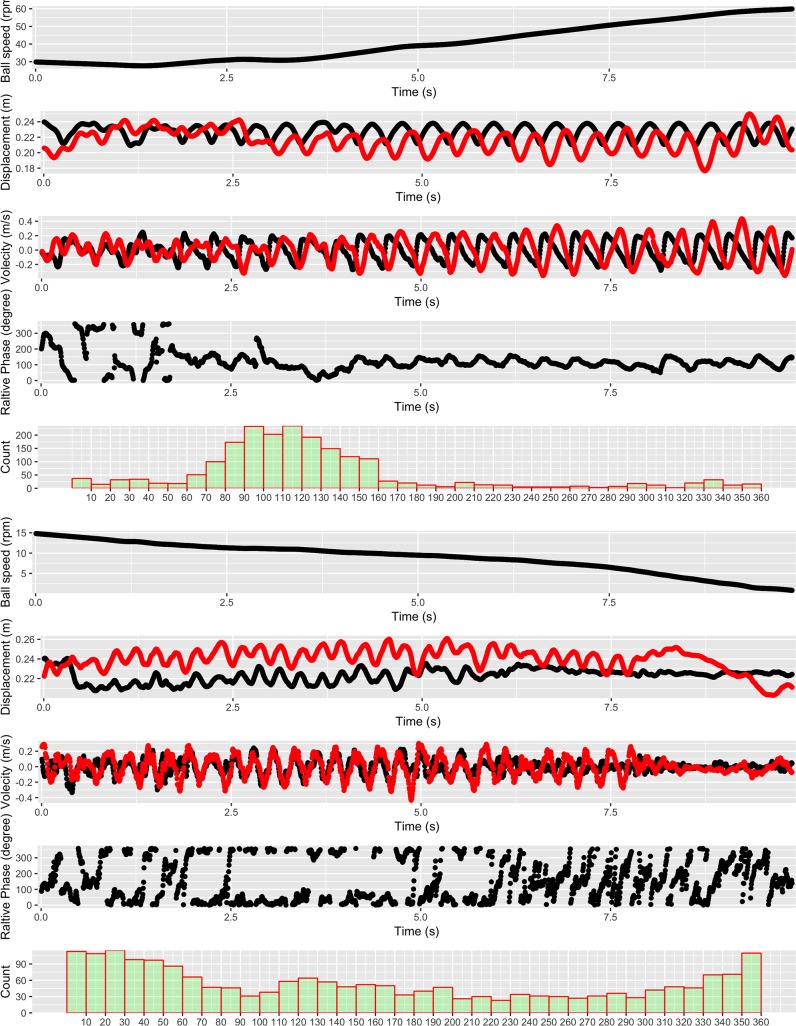
**The roller ball movement performance of example success trial (top panel) and failure trial (bottom panel).** There are 5 performance measures in each trial: from top down, roller ball speed profile, displacement of the outer shell (red/light) and inner top (black/dark), velocity of the outer shell (red/light) and inner top (black/dark), relative phase of the outer shell and inner top, relative phase distribution from 0 to 360 degrees.

Fig 6 shows single set of trial time series of movement characteristics for a successful (top segment) and a failed (bottom segment) performance. The time series clearly show the random-like initial search for the collective variable and the subsequent synchronous motion of ball and outer shell. The transition point of a drop in ball speed to an increment in ball speed was related in time to the formation of the synchronous motion of the collective variable.

**Fig 7:**
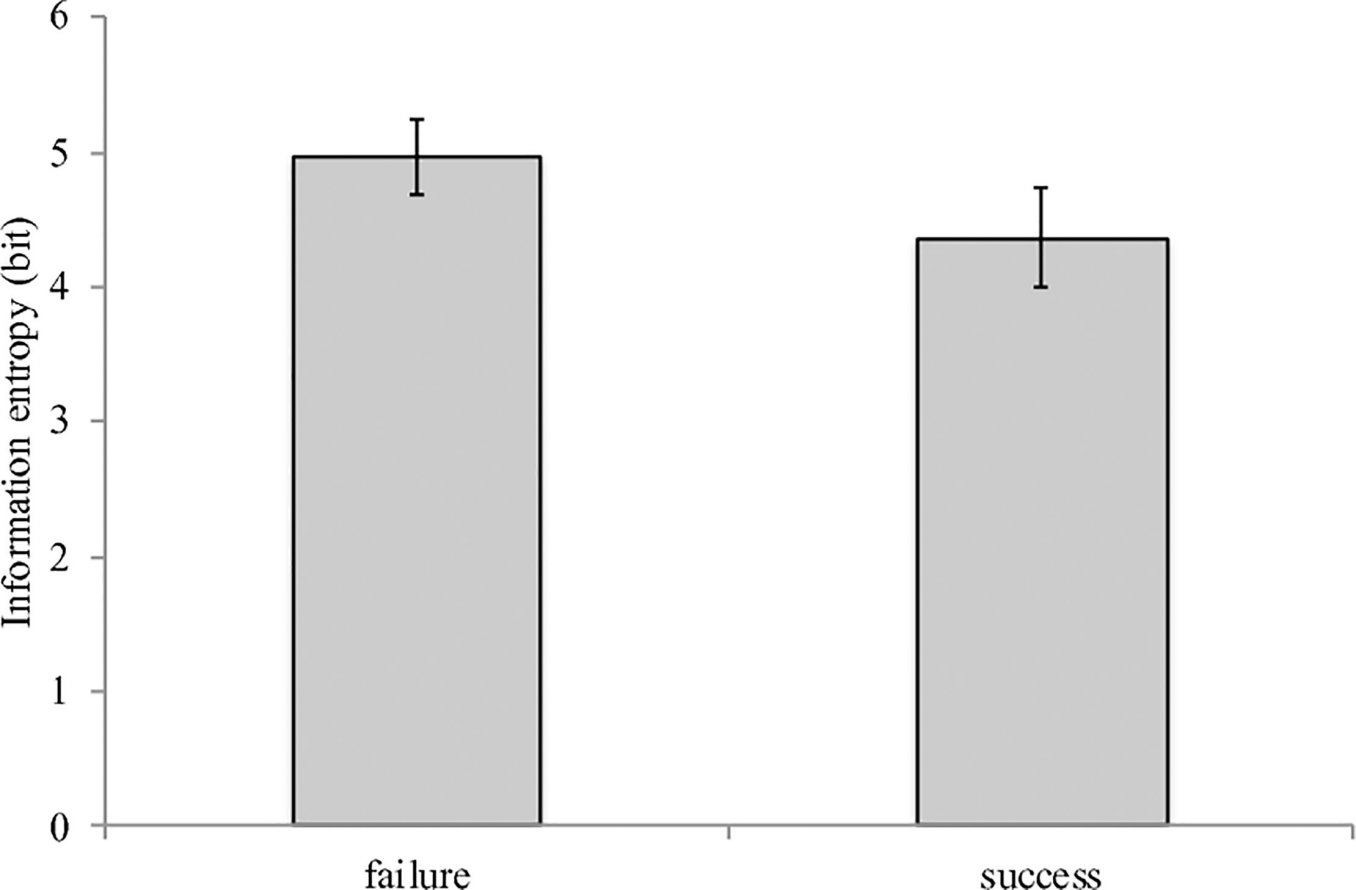
The average information entropy of the relative phase for the success trials and the failure trials. The error bars indicate the standard deviation over the 16 participants.

A *t* test on information entropy, that measures the level of variability of the relative phase, was performed to examine the successful and failure trials. Thirty-six bins were used in calculating the distribution over the 360 degrees of relative phase. The *t* test showed a significantly lower information entropy for the success trials than the failure trials, *t*_*15*_ = 8.74, *p* < .001, *Cohen’s d* = 2.12 (Fig 7). The lower information entropy of the relative phase from the successful trials indicates a more concentrated distribution of the relative phase, that is, more stable relative phase as a result of the synchronous motions between the outer shell and the inner ball.

### Synergy

**Fig 8:**
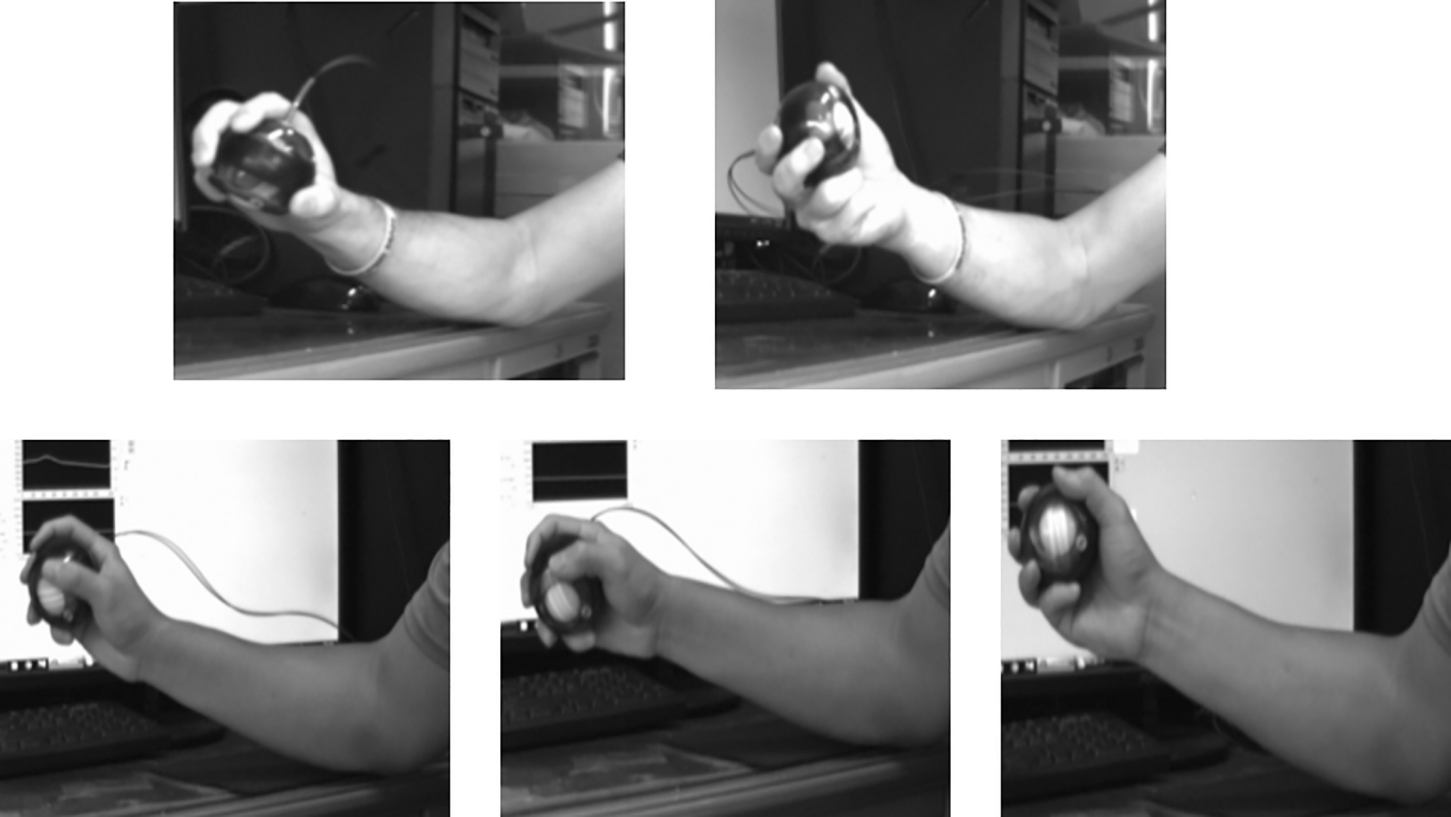
Two basic roller ball movement patterns observed from the experiment. The top 2 pictures show the pattern of pronation-supination of the proximal radio-ulnar joint only; the bottom 3 pictures depict the movement pattern composed of proximal radio-ulnar joint and the wrist rotation movement. The bottom pattern was observed rotating in both clockwise and counterclockwise directions.

Visually examining the 800 trials of the roller ball task from the video recordings revealed there were 2 basic categories of the arm-hand movement patterns for the roller ball task: pronation-supination of the proximal radio-ulnar joint only (type 1) or the radio-ulnar joint movement together with wrist rotation (type 2)(see Fig 8). The wrist rotation patterns could be further categorized into clockwise (type 2c) and counterclockwise (type 2cc).

Nine participants performed 100% trials with one movement pattern, five participants had 6% to 8% of trials performed with different movement patterns from their main patterns. Among these fourteen participants, five participants used mainly type 1 movement pattern, three participants used mainly type 2c movement pattern, and six participants used mainly type 2cc movement pattern. Two participants had similar percentage of trials performed with 2 different movement patterns, one of them used type 1 and type 2c and the other one used type 2c and type 2cc. There were six participants performed 2%-18% trials with two movement patterns in a trial. The ANOVA on the outer shell movement frequency showed no difference among the 3 movement patterns, *F*(2, 107) = 1.98, *p* > .05, η_p_^2^ = .04, the mean (SD) frequency for type 1, type 2c, and type 2cc was 3.82 (1.14) Hz, 3.47 (0.66) Hz, and 3.40 (1.07) Hz, respectively.

## Discussion

The purpose of the present study was to investigate the mapping of collective variable and synergy dynamics with task outcome within the general context of the multi-layered framework of complex adaptive dynamical systems [2–6]. In particular, we examined the effect of a control parameter, namely, the initial condition of ball speed in the roller ball task, on short-term adaptation in task dynamic properties of the perceptual-motor system. The perceptual-motor properties analyzed were task outcome, the candidate collective variable (inner ball-outer shell relative motion) and neuromuscular synergies of the arm-hand kinematic chain.

### Task outcome

The findings were clear in showing a strong adaptive effect of initial ball speed on task success [17–18] that was defined as the ball speed reaching above the initial ball speed condition for the respective trial. Indeed, the task performance ranged from zero trial success in all participants at the lowest initial ball speed of 10 rps (where no participant was able to increase ball speed from the initial speed) that progressed to a 100% success for all individuals at the higher initial ball speeds of 30 and 40 rps. This finding is consistent with the view that the system changed from a highly stable but failure state as a task-relevant solution at the slow initial ball speeds to highly stable success state at the high initial ball speeds.

The increment of short-term adaptive performance from the limited trial practice structure used here was not linear with initial ball speed. It was not a main focus of the experiment to determine this performance function–hence, the limited ball speed conditions. Nevertheless, the task success data (Table 1), when considered in conjunction with the slope of the increment in ball acceleration (Fig 4), point to a sigmoid function for the effect of initial ball speed on task performance. These findings on the difficulty of the initial ball speed condition manipulation are consistent with those of our earlier studies [17–19] on S-shape learning where practice time was considered as the control parameter to elicit a successful outcome. Furthermore, they provide the basis to interpret the new findings obtained here of the parallel adaptive features at other task properties of the system organization in the roller ball.

The ball speed trajectory that reflected the within-trial performance was analyzed in addition to the qualitative task outcome of success or failure of ball speed determined at the single point in time at the end of the trial. Within a trial, the ball speed showed an initial decrease prior to a transition and subsequent increment that was dependent on the initial ball speed condition. Thus, we characterized the total within trial task performance of the ball speed trajectory into 2 sequential components.

There was an initial perceptual-motor search component [39] of the inner ball-outer shell relative motions to form the relevant collective variable for the task that was followed by the stabilization of the inner ball-outer shell synchronous motion that facilitates the progressive increase in ball speed. The initial search component was characterized by the duration of this initial drop in ball speed at the beginning of a trial and was shown to be longer in the lower initial speed conditions indicating a longer search time to form the task relevant collective variable of the synchronous relation of inner ball to outer shell frequency.

The effect of ball speed on this search process is, we hypothesize, related to the haptic feedback that mediates the motion of the arm-hand kinematic chain particularly in the enhanced precession force arising during the high initial ball speed conditions. When the initial ball speed is low, it could be that the resulting slow precession provides weak haptic feedback to the arm-hand system through the motion of the outer shell; therefore, a longer search duration is needed for generating a synchronous outer shell to inner ball motion. Failure to form the synchronous outer shell to inner ball motion during the initial search duration will result in a continuing decrease of the ball speed and the eventual failure in the task.

The second component of the performance trajectory where the ball speed starts to increase was also influenced by the initial ball speed. The low initial ball speed not only takes a longer initial search duration due to the weak haptic feedback of the slow precession, it also has lower acceleration for the ball spinning. The synchrony between the outer shell and the inner ball may be intermittent due to the weak haptic feedback that hampers the steady increase of the ball speed. This indicates that the rate of stabilization of the collective variable was influenced by the initial ball speed acting as a control parameter. When the value of the control parameter increases, the search duration of the synchrony between the outer shell and inner ball motions is shortened due to the stronger precession force from the initial ball speed, which leads to the strengthening of the spinning roller ball attractor state. On the other hand, the value of control parameter decreases, the diminishing precession force from the low initial ball speed may result in an increased search duration for the synchrony that leads to an eventual stop of the inner ball. In the middle range of the control parameter, the dynamical state of the roller ball may fluctuate between spinning and stopping depending on the performer’s skill level (practice experience).

### Synchrony of inner ball and outer shell motion as the collective variable

The findings provide strong evidence that the key task dynamic property for a successful trial is the synchrony of the respective frequencies of the motions of the inner ball and outer shell. The analysis of the matching frequencies mapped well with, respectively, success and failure in the task (Table 2). This role for frequency coupling rather than relative phase as the collective variable in this continuous, rhythmic roller ball task is because the motion of the ball is not restricted to a particular spatial position within the shell as would be the case with a relative phase relation. When the shell and the ball are matched in frequency, however, a consistent relative phase between the two parts would be observed given any fixed pair of points on the system. These contrasting findings on the collective variable are consistent with coordination dynamics that the collective variable is to be determined from the interactions of the constraints to action [3].

The information entropy analysis on the distribution of the relative phase showed that the entropy of the failure trials was significantly larger than that of the successful trials, further supporting the synchrony of the outer shell-ball relation as the collective variable in the roller ball task. This is one way in which the roller ball task provides a different and richer set of constraints to the coupling of the dynamics of the inner ball motion and the product of the arm-hand movement system (the outer shell motion) than the more restricted constraints on performing or learning a given relative phase in bimanual finger control [16]. More generally, it reflects an example of the specific influence of task constraints in the determination of the collective variable that is based on the mechanical principles of the task.

The roller ball task allows the separation of the collective variable from the neuromuscular synergies of particular joint motions that drive the movement sequence–variables that are in essence the same in the bimanual finger coordination task [7]. The task goal of many other motor activities, such as basketball shooting or hula hooping, are not defined by the coordination of the body-limbs system but are determined by the interaction between the coordination of the body-limbs system and an external object or property of the environment. An understanding of the task dynamics of these types of tasks would involve comprehensive analysis of the movement system of the actor in conjunction with that of an object property [23]. The current study provides an example for examination of a fuller range of the related levels of task dynamics than the body-limb coordination tasks [33–34].

Considering performance in the roller ball task as that arising from a complex adaptive system, allows for and anticipates the adaptive contributions of degeneracy [32] to the properties of the task dynamics. The different characterizations of degeneracy between the neuromuscular synergy and the collective variable and between the collective variable and the task outcome is, we conjecture, typical of many perceptual-motor skills considered in context of a complex adaptive system [5].

### Neuromuscular synergies

We were able to separate the motions of the neuromuscular arm-hand synergies from the motions of the collective variable in the roller ball task. Two types of neuromuscular synergy structures were identified in execution of the task: namely, the pronation-supination of the proximal radial-ulnar joint and this radio-ulnar joint motion with the addition of wrist motion [23–24]. The latter category was further separated into clockwise and counter-clockwise motions of the hand to create three qualitatively distinct neuromuscular synergy motions used by participants in the execution of the roller ball task. These three synergy motions accounted for most of the variance in the qualitative dynamics of the neuromuscular system in performing the roller ball task.

The majority of participants used only one of the arm-hand neuromuscular synergies while two participants used two of the synergies in approximately even numbers. Significantly, the prevalence of the three synergetic movement patterns was not related to the initial ball speed condition or whether the trial was a success or failure at the task level. This finding supports the interpretation that the participants brought to the roller ball task particular neuromuscular synergy preferences (intrinsic dynamics) but given the degeneracy afforded by the confluence of constraints each of these synergies could satisfy the task demands. Whether further practice or different sets of task constraints would lead to a different profile or effect of neuromuscular arm-hand synergy use is an empirical question. McDonald, Oliver and Newell [40] showed that the search strategies arising from within- and between-limb neuromuscular synergies followed similar principles.

### Mapping between task outcome, collective variable, and synergy

The findings provide strong evidence for the adaptive mapping of task outcome, the collective variable of inner ball–outer shell synchronous motion, and neuromuscular arm-hand synergies. This mapping, however, is not of a one to one relation of within-trial dynamics at each of the three properties of task dynamics examined. Rather, there is variability and degeneracy of the solutions within and between the synergies and the collective variable and between the collective variable and task outcome [32].

A critical process in performing the roller ball task successfully is the search for the formation of the collective variable: namely, the task relevant synchronous motion between the inner ball and outer shell. Indeed, this synchronous variable seems to be the critical dynamic feature to task success in the roller ball. The short-term adaptation to the roller ball task as a function of ball speed affords the operational distinction of the three variable properties of task outcome, collective variable and neuromuscular synergy [33–34]. These categories of variables reflect the distinction from Bernstein [41] of the synergy, space and action levels of control. Furthermore, the control variable of ball speed was the factor that determined success and failure in the task and allowed us to investigate the mapping of categories of task dynamics under different stability regimens.

In the framework of coordination dynamics there is reciprocal causality between the collective variable and the motions of the individual components and synergies [3]. The roller ball task with its capacity to separate the collective variable from the neuromuscular synergies affords the potential of a representative test of this hypothesized mapping across task variables. Indeed, the integrated findings of the task dynamics provide support to the view that an important direction for research in motor control and skill acquisition is the unpacking of the collective variable across the varieties of perceptual-motor skills.

Most experiments in motor learning and control use a task such as mirror tracing and limb positioning/timing in which the participants can already form a task-relevant collective variable and coordination mode leaving the adaptive scaling of the task outcome as reflected in the functions of exponential and power law learning curves [42, 43]. However, in tasks that require the acquisition of a new (that is, previously unperformed) coordination mode the formation of the qualitative nature of the task relevant coordination mode is essential in the very early stage of learning [44]. Indeed, the theoretical construct of collective variable provides a principled way to examine the evolving formation of new patterns of movement coordination [44], regardless of whether the task goal is the movement pattern per se, or a particular outcome from the movement pattern–context interaction.

Finally, characterizing the emergence of the collective variable and its relation to other properties of task dynamics may also be a way to formalize from a dynamical perspective of Fitts’ [45] notion of ‘getting the idea of the task’ in the early stage of motor learning [46]. The emergence and formation of the coordinative structure early in learning has not been an issue in the motor skill domain because until the introduction of the dynamics of coordinative structures [3,6] there has been no theoretical framework to investigate such phenomena. And, even if the relevant stimulus of theory had been present earlier to guide experimentation on movement coordination, it would have required the learning of tasks different than the usual candidates of choice for motor learning. Motor learning experiments have typically finessed investigation of the essence of Bernstein’s [41] DF problem by using tasks where the participant can already produce the dynamics of the collective variable on the initial trial(s) of the practice session [44]. In short, understanding the influence of task dynamics on coordination and control of movement and posture requires a fuller set of the available DFs to be examined if the general Bernstein problem of skill as the mastery of redundant DFs is to be decomposed and understood.

## Supporting information

S1 Dataset Performance data of each participant. (XLSX)

S2 Dataset Data of the selected 113 trials. (XLSX)
